# Antibiotic Use in a Municipal Veterinary Clinic in Ghana

**DOI:** 10.3390/tropicalmed6030138

**Published:** 2021-07-20

**Authors:** Wisdom Adeapena, Samuel Afari-Asiedu, Robinah Najjemba, Johan van Griensven, Alexandre Delamou, Kwame Ohene Buabeng, Kwaku Poku Asante

**Affiliations:** 1 Kintampo Health Research Centre, Ghana Health Service, Kintampo North Municipality, Kintampo P.O. Box 200, Ghana; samuel.afari-asiedu@kintampo-hrc.org (S.A.-A.); kwakupoku.asante@kintampo-hrc.org (K.P.A.); 2 Public Health Consultant, 1202 Geneva, Switzerland; robinahnajjemba@yahoo.co.uk; 3 Institute of Tropical Medicine, B-20000 Antwerp, Belgium; jvangriensven@itg.be; 4 Africa Centre of Excellence for Prevention and Control of Transmissible Diseases (CEA-PCMT), Gamal Abdel Nasser University of Conakry, Maferinyah 4099, Guinea; adelamou@gmail.com; 5 Department of Pharmacy Practice, Faculty of Pharmacy and Pharmaceutical Sciences, College of Health Sciences, Kwame Nkrumah University of Science and Technology, Kumasi P.O. Box 93, Ghana; kobuabeng.pharm@knust.edu.gh

**Keywords:** antimicrobial resistance, antibiotic use, veterinary service, SORT IT, operational research

## Abstract

Antimicrobial resistance (AMR) is a threat to public health, impacting both human and animal health as well as the economy. This study sought to describe antibiotic prescription practices and use in the Kintampo North Municipal Veterinary Clinic in Ghana using routinely collected data. Of the 513 animals presented for care between 2013 and 2019, the most common animals were dogs (71.9%), goats (13.1%), and sheep (11.1%). Antibiotics were prescribed for 273/513 (53.2%) of the animals. Tetracycline was the most commonly prescribed class of antibiotics, (99.6%). Of the 273 animals that received antibiotics, the route of administration was not documented in 68.9%, and antibiotic doses were missing in the treatment records in 37.7%. Details of the antibiotic regimen and the medical conditions diagnosed were often not recorded (52.8%). This study recommends appropriate documentation to enable continuous audit of antibiotic prescription practice and to improve quality of use. There is also the need for a national survey on antibiotic prescribtion and use in animal health to support policy implementation and decision making in One-Health in Ghana.

## 1. Introduction

Antimicrobial resistance (AMR) is an increasing threat to public health and advances in the fight against infectious diseases [1,2]. Antibiotic use in humans, animals, and agriculture, is one of the drivers of AMR. Recently, animal production practices have been associated with regular antibiotic use, and foodborne transmission of antibiotic-resistant bacteria from animals to humans is well documented [3,4]. Global consumption estimates suggest that antimicrobial use in animals is twice that of humans [5].

While trends in antibiotic consumption in humans are increasingly being tracked globally, antibiotic consumption in livestock and the veterinary service has received comparatively little attention [6]. There is limited information on antibiotic use in animals in rural veterinary services in West Africa and none from Ghana [7,8]. International guidelines of the World Organization for Animal Health (OIE) recommend implementing a surveillance system for antibiotic consumption within veterinary services, including information on the antibiotic class used, the total dose, and the route of administration [9]. However, the extent to which such systems are currently implemented in low- and middle-income countries is unclear [10].

The Ministry of Health (MOH), Ghana, has developed Standard Treatment Guidelines to aid the appropriate use of medicines, including antibiotics [11]. These guidelines help health professionals in the selection of medicines for treating infectious diseases in humans. There are no guidelines for the prescription of antibiotics in animal health [12,13]. The Veterinary Public Health and Food Safety Unit together with the Laboratory and Epidemiology Units of the Veterinary Services Division (VSD) are responsible for monitoring the use of antibiotics and surveillance of resistance in animals [14]. According to the Public Health Act of Ghana, 851, 2012, the Food and Drugs Authority is responsible for regulating the importation and registration; post market surveillance of all medicines, including antimicrobials, used in veterinary services. The activities of these regulatory bodies are meant to support the surveillance of AMR.

To holistically address the challenge of AMR, WHO adopted the Global Action Plan (GAP-AMR) in 2015 based on the concept of “one health” to implement strategies through relevant sectors and stakeholders. It is expected that the use of antimicrobial agents in humans, plants, and animal health would be optimized in the ‘one health’ approach through specific interventions to promote the responsible use of antimicrobials in humans, veterinary, and aquaculture as well as in the environment and industry [14]. In line with GAP, Ghana through the national AMR platform has developed an AMR policy and is currently implementing a National Action Plan to fight AMR [14,15]. The policy recommends specific regulatory interventions to deal with manufacturing and the supply chain of antimicrobials. The veterinary service under the Ministry of Agriculture is one of the key stakeholders of the AMR platform and one of their objectives is to carry out surveillance to provide baseline data on AMR use in animal husbandry and veterinary care. Understanding the context of antibiotic prescription practices will inform the development of targeted intervention to improve use within veterinary services in Ghana.

This study explored (a) the number and type of animals receiving veterinary care, (b) proportion of animals receiving antibiotics by type of animal, (c) the prescription practices (documentation of antibiotic class, dosage, and route of administration), and (d) the type of antibiotics used at Kintampo Municipal Veterinary Clinic, Ghana, from 2013 to 2019.

## 2. Materials and Methods

### 2.1. Study Design

This was a descriptive study using routinely collected secondary data from the treatment records at the Kintampo Municipal Veterinary Clinic.

### 2.2. Study Setting

#### 2.2.1. General Setting

The livestock sector contributes to about eight percent (8%) of Ghana’s Gross Domestic Product (GDP) [16]. Veterinary services are under the regulation of the Veterinary Service Directorate, which is under the Ministry of Food and Agriculture. Public and private animal health care facilities are found mostly in towns and cities, with a few in rural areas. There are approximately 256 veterinary clinics nationwide. Clients to these facilities in the urban areas are mostly owners of pets, such as dogs, cats, and monkeys [17]. Small-scale livestock farmers rely mainly on veterinarians or other animal health workers from the public sector [8,15].

The Veterinary Council of Ghana is responsible for supervising veterinary doctors. There is a national information management system that collates monthly and quarterly reports from the district officers through the regional directorate. Every district has a District Veterinary Officer and several veterinary paraprofessionals. Currently, there is no national electronic database, so that all the reports are sent in as hard copies [18]. The government monitors the provision of veterinary medicines through a centralized system with the support of private veterinary pharmaceutical entities. The Veterinary Service Directorate (VSD) has the responsibility of ensuring provision of veterinary drugs and vaccines through both public and private procurement systems. Drug management at the VSD is in compliance with the Economic Community of West African States (ECOWAS) veterinary pharmacy protocol [13,19]. These drugs are distributed to the various regional veterinary directorates and subsequently to the districts and other private veterinary medicine outlets across the country.

#### 2.2.2. Specific Setting

**Figure 1:**
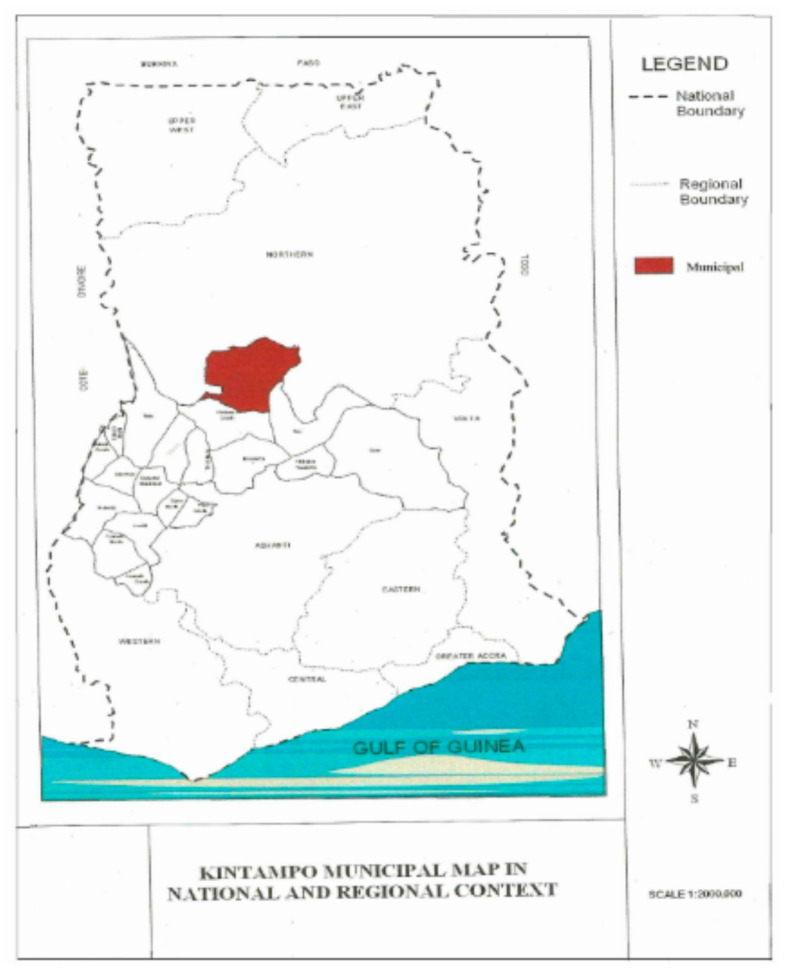
Map of Ghana showing Kintampo North Municipal.

The Kintampo North Municipality is located within the forest-savannah in the middle belt of Ghana (Figure 1). The human population of the Kintampo Municipality, as at April 2020, was 163,172 [20]. The major livestock species kept as at 2014 were cattle (1,657,000), sheep (4,335,000), goats, (6,044,000), pigs, (682,000), and 68,511,000 poultry. Most consumed livestock were cattle, goats, sheep, and pigs. Generally, over 70% of the population are involved in agriculture (plant and animal). There is a commercial goat, pig, and bird breeding station, which serves as a market for animal farmers. Livestock farming activities in the area are on the increase, and there is a greater dependence on them as a source of employment and meat production [16].

The Kintampo Municipal Veterinary Clinic was selected for this study because it is the only clinic providing veterinary services in the study area. Though the clinic is situated in the municipality, its catchment area includes more than 100 rural communities. The size of these rural communities range from 40 compounds to approximately 1500 compounds [20]. As of 2018, the clinic had one veterinary doctor and one veterinary technician who provided routine clinical and surgical services for both pets and farm animals. The number of staff increased to nine in 2020 with two clinicians and seven field operation officers. The clinic has had two changes in management between 2013 and 2019, and the current chief clinician has been at the post since 2019. The clinic lacks animal laboratory services, so diagnoses are based on the clinical experience of the staff. The owners of the animals either bring them to the clinic or are visited by the veterinary field staff in their communities. As of March, 2021, the cost of non-surgical treatment per animal visit was between GHC15.00 and GHC20.00 (USD 2.61–USD 3.48). Medicines used for treatment are stocked at the clinic. However, when they are out of stock, purchases are made from private veterinary medicine outlets. Apart from clinical and surgical services, staff of the clinic carry out community sensitization and vaccination programs on an annual basis to educate the community on zoonotic diseases and domestic and farm animal care. On the local front, there is unregulated access to veterinary drugs [18] via one private veterinary medicine outlet.

There is a standard treatment register at the clinic where data on the management of the animals is recorded. It includes the type of animal, breed, diagnosis, dosage of medicine (if prescribed), and route of administration. Quarterly reports are sent to the regional directorate as part of a monitoring and evaluation system.

### 2.3. Study Population

All animals treated in the Kintampo Municipal Veterinary Clinic from 2013 to 2019 were included in the study.

### 2.4. Data Variables and Sources of Data

Data were extracted from the treatment register of the Kintampo Municipal Veterinary Clinic, including details, such as the type of animal (dog, goat, sheep, cat, cow, chicken, pig, rabbit, monkey), date of treatment, condition for which the animal was treated, and use, type, dosage, and route of administration of antibiotics if they were prescribed. The principal investigator extracted the data, which were then double entered by two senior data entry clerks. However, due to missing data, treatment records from 2016 and 2017 were not included in the study.

### 2.5. Data Entry and Analysis

Data were entered into an Excel spreadsheet and imported into Stata 14 for analysis. The number and percentage of various types of animals treated across 2013 to 2015 and 2018 to 2019 were described. The number and proportion of animals treated with antibiotics were calculated across the study years. Similarly, the number, dosage, and route of antibiotics administered were calculated.

## 3. Results

### 3.1. The Number and Proportion of Animals Receiving Veterinary Care by Type of Animals

In total, 513 animals were presented for treatment during the study years (2013 to 2015, 2018, and 2019), (Table 1). The most commonly treated animals were dogs (71.9%), followed by goats (13.1%) and sheep (11.1%) with the rest constituting (3.9%). The least commonly treated animals were rabbits (2) and monkeys (1). The number of animals presenting at the clinic decreased from 135 in 2013 to 78 in 2015. The lowest number was seen in 2018 (40 animals), but this number increased to 149 in 2019.

**Figure 2:**
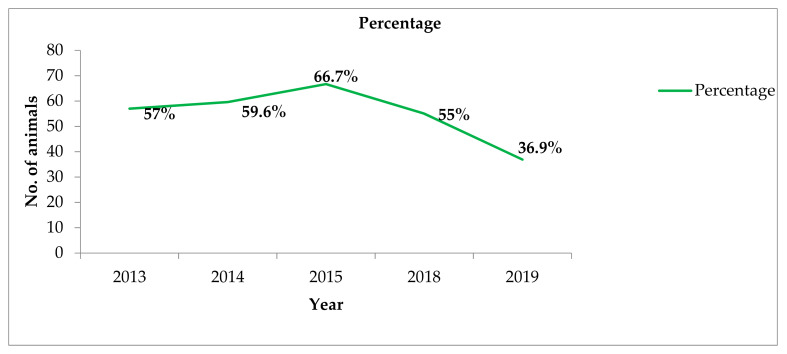
Proportion of antibiotic use among animals receiving care from the Kintampo Municipal Veterinary Clinic in Ghana stratified by year, 2013–2019. The clinic changed location in 2019 and records for 2016 and 2017 were lost, thus data were not available for review.

In 2013, 57% of animals received antibiotics compared to 36.9% in 2019. There was a decrease (55.0% to 36.9%) in the use of antibiotics between 2018 and 2019 (Figure 2).

### 3.2. The Number and Proportion of Animals Receiving Antibiotics by Type of Animal and Year

**Figure 3:**
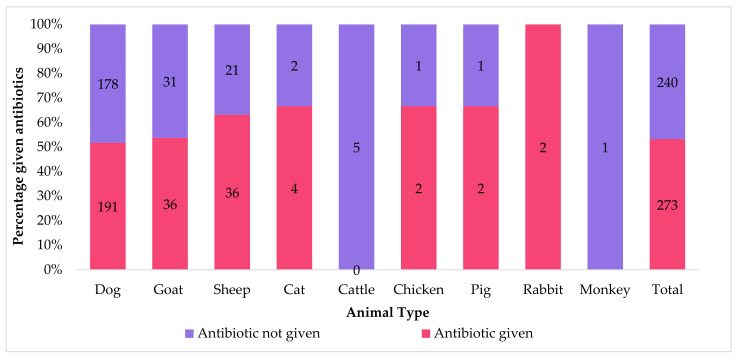
Number and proportion of antibiotic use by type of animal that received antibiotics from the Kintampo Municipal Veterinary Clinic in Ghana, 2013–2019.

Of 513 animals, more than half received antibiotics (273, 53.2%). For the most common animals (dogs, goats, and sheep) antibiotic prescriptions ranged from 51% to 63%, as shown in Figure 3.

### 3.3. The Documentation of Condition, Antibiotics Prescribed, Dosage, and Route of Administration

**Figure 4:**
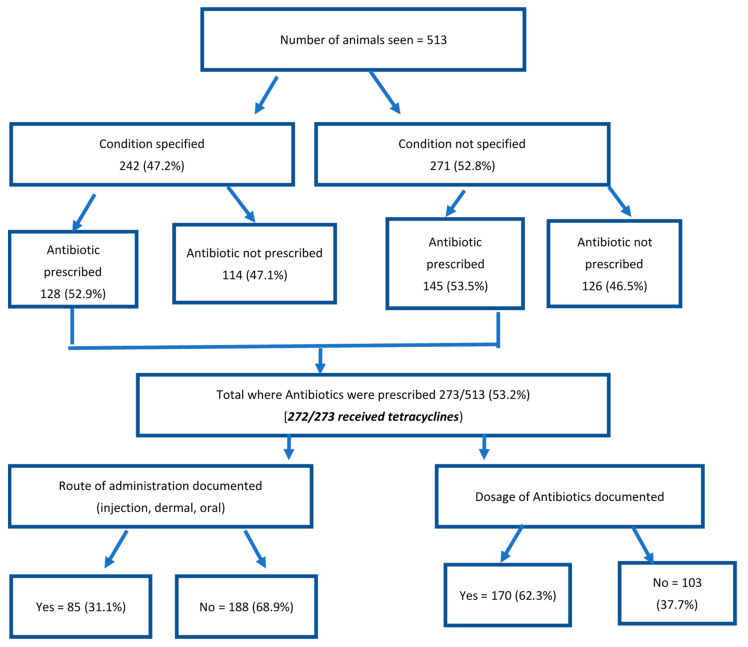
Flow chart depicting the specification of disease conditions and the documentation of antibiotics dosage and route of administration in the Kintampo Municipal Veterinary Clinic in Ghana, from 2013–2019.

The tetracycline class of antibiotics (272 out of 273) was almost exclusively used, while penicillin was used for only one animal. Of the 273 animals receiving antibiotics, the route of administration was not documented in the majority (188, 68.9%) and the details of antibiotic dosage were missing in the treatment records for one in three animals (103, 37.7%). Of the 85 (31.1%) animals in whom the route of administration was mentioned, injection was the most common (46, 54.1%), followed by dermal application (32, 37.6%) and oral (7, 8.2%). About 271 (52.8%) of the disease conditions were not specified out of which 145 (53.5%) were still treated with antibiotics (Figure 4).

## 4. Discussion

Antibiotics were prescribed to more than half of the animals that presented for care; virtually all were tetracycline. Considerable gaps in documentation, including failure to record diagnosis, dosage of antibiotic, and route of administration, were observed.

Our findings on the considerable use of tetracycline to treat animals are similar to studies conducted in Rwanda and Ghana among animal farmers and veterinary care providers, where oxy-tetracycline was commonly used [5,21]. This finding may be a result of the lack of national standard treatment guidelines in Ghana for the prescription of antibiotics in the veterinary service [14] as found in other Asian and European countries [22,23]. Though there is a national drug policy regulating the use of all medicines in Ghana, the provisions in the policy do not adequately provide for the control of antibiotic use in veterinary service [12,24]. Additionally, there is no separate policy or document to address the issues of antibiotic use in animal health under the One Health concept as proposed by the WHO. The predominant use of tetracycline could be attributed to its ability to treat varied infections in animals [9,25]. Additionally, given that the study setting is rural, there could be an inadequate supply of drugs and logistics for laboratory services, which could account for the reliance on the experience of veterinary care providers. The Veterinary Service Directorate has the responsibility of ensuring the provision of veterinary drugs and vaccines through both public and private procurement systems. Drug management at the VSD is in compliance with the Economic Community of West African States (ECOWAS) veterinary pharmacy protocol [13,19]. These drugs are distributed to the various regional veterinary directorates and subsequently to the districts and other private veterinary medicine outlets across the country. The ease of access to these drugs, especially antibiotics, in over the counter medicine sellers (OTCMSs) could promote misuse [2,26].

The WHO recently classified veterinary antibiotics according to their risk to compromise antibiotic treatment for humans [9,25,27,28,29]. According to this classification, tetracyclines are classified as a highly important human medicine and strict monitoring of their use in animals is recommended [30]. Future studies are recommended to assess whether antibiotic prescriptions are justified and whether other antibiotics with a lesser impact on human health could be used. That said, the predominant use of tetracycline in this study and other studies in Ghana [31] calls for the development of a national antibiotic treatment guideline for animal health [32].

Incompleteness in the recording of the diagnosis, antibiotic dosage, and route of administration was observed. This finding is similar to inadequate documentation practices found in human [33] and animal [34,35] health care within developing countries. The observation of the shortfall in documentation may be attributed to the lack of adequate training on structured systems of recording data on antibiotics use in animal health as specified in the protocols of the OIE, which are used to monitor factors that influence antibiotic resistance patterns [9,36]. Improved systems, such as electronic tools, for documentation and tracking of antibiotics prescription and use practices by the veterinary services will be useful as reported in other countries [37]. Regular and comprehensive data on antimicrobial use in veterinary medicine could contribute to improved prescribing practices.

There are some important limitations to our study. First, we could not fully capture antibiotic prescription trends across the entire study period as data were missing for two years. Second, data on the medical conditions among the animals receiving antibiotics were inadequate as they were either missing or lacked sufficient detail. Hence, it was not possible to gauge to what extent antibiotic prescriptions were justified. Finally, we cannot generalize the findings to other settings as we only collected data from one veterinary clinic. Notwithstanding, our findings highlight the need for a broader national survey to further assess antibiotic prescription practices in the veterinary service as an important step for decision-making by key stakeholders, such as the Ghana National AMR platform.

## 5. Conclusions

Antibiotics, specifically tetracycline, were commonly used for the treatment of animals in one veterinary clinic in Ghana within the context of poor documentation of antibiotic prescription and use practices. There is a need for a national survey to further assess antibiotic prescription and use practices to support decision-making by stakeholders in the fight against antimicrobial resistance in Ghana and other low- and middle-income countries.

## Figures and Tables

**Table 1:** Number and type of animals that received veterinary care at the Kintampo Municipal Veterinary Clinic in Ghana stratified by year, 2013–2019.

Type of Animal	2013		2014		2015		2018		2019		Total	
	**n**	**(%)**	**n**	**(%)**	**N**	**(%)**	**n**	**(%)**	**n**	**(%)**	**n**	**(%)**
Dog	96	(71.1)	76	(69.7)	61	(78.2)	26	(65.0)	108	(72.5)	369	(71.9)
Goat	24	(17.8)	17	(15.6)	5	(6.4)	7	(17.5)	14	(9.4)	67	(13.1)
Sheep	11	(8.1)	12	(11.0)	9	(11.5)	4	(10.0)	21	(14.1)	57	(11.1)
Cat	4	(3.0)	0	(0.0)	0	(0.0)	1	(2.5)	1	(0.7)	6	(1.2)
Cow	0	(0.0)	2	(1.8)	0	(0.0)	0	(0.0)	3	(2.0)	5	(1.0)
Chicken	0	(0.0)	1	(0.9)	1	(1.3)	1	(2.5)	0	(0.0)	3	(0.6)
Pig	0	(0.0)	1	(0.9)	2	(2.6)	0	(0.0)	0	(0.0)	3	(0.6)
Rabbit	0	(0.0)	0	(0.0)	0	(0.0)	1	(2.5)	1	(0.7)	2	(0.4)
Monkey	0	(0.0)	0	(0.0)	0	(0.0)	0	(0.0)	1	(0.7)	1	(0.2)
**Total**	**135**	**(100.0)**	**109**	**(100.0)**	**78**	**(100.0)**	**40**	**(100.0)**	**149**	**(100.0)**	**513**	**(100.0)**

The clinic changed location in 2019 and in the process, records for 2016 and 2017 were lost, thus data was not available for review.

## Data Availability

Generated datasets for this study are available upon reasonable request to the corresponding author either in hard or soft copy. Also contact can be made to the Kintampo Municipal veterinary clinic for the availability of the data.
